# Psychosocial functionning in euthymic bipolar patients

**DOI:** 10.1192/j.eurpsy.2021.513

**Published:** 2021-08-13

**Authors:** S. Elleuch, R. Sellami, R. Ouali, R. Masmoudi, I. Feki, J. Masmoudi

**Affiliations:** Psychiatrie “a” Department, Hedi Chaker Hospital University -Sfax - Tunisia, sfax, Tunisia

**Keywords:** quality of life, bipolar disorder, FAST scale, euthymia

## Abstract

**Introduction:**

Bipolar disorder (BD) is a chronic, recurring illness that can lead to serious disruptions in functioning.

**Objectives:**

To evaluate functionning in this population and to explore the relationship with socio-demographic and clinical features of BD.

**Methods:**

This is a descriptive and analytical cross-sectional study including patients with BD (DSM V) in euthymia, followed on an ambulatory basis to the Mood Disorders Unit of the Psychiatry A Department at Hedi Chaker Hospital University of Sfax between January and April 2019. Patients were considered euthymic if they scored less than 7 on the Young Mania (YMRS) rating scale and less than 8 on the Hamilton Depression scale (HDRS-17). The Short Function Evaluation Test (FAST scale) was used to evaluate functionning. Global functional impairment is defined by a total FAST score>11.

**Results:**

We recruited 62 patients with a mean age of 45.65 years (SD=13.3) and a sex ratio of 1.13. 88.7% of patients were followed for BD I and 11.3% for BD II. The mean age of onset was 29.37 years (SD=11.6). The mean numbers of manic and depressive episodes were respectively 3.73 (SD=3.8) and 2.48 (SD=2.9). The mean FAST score was 28.97 (SD=15). Overall impairment was observed in 85.5% of patients. Impaired functionning was significantly more frequent in patients with a history of surgery (p=0.046), in those with a higher number of depressive episodes (p<0.001) and in subjects with partial remission (p=0.01).

**Conclusions:**

Thus, the treatment should target not only the improvement of symptoms but also the reduction of the incapacity of patients.

